# An in-field heat treatment to reduce *Cercospora beticola* survival in plant residue and improve Cercospora leaf spot management in sugarbeet

**DOI:** 10.3389/fpls.2023.1100595

**Published:** 2023-05-09

**Authors:** Alexandra P. Hernandez, Daniel M. Bublitz, Thomas J. Wenzel, Sarah K. Ruth, Chris Bloomingdale, David C. Mettler, Mark W. Bloomquist, Linda E. Hanson, Jaime F. Willbur

**Affiliations:** ^1^ Department of Plant, Soil and Microbial Sciences, Potato and Sugarbeet Pathology, Michigan State University, East Lansing, MI, United States; ^2^ Department of Plant, Soil and Microbial Sciences, Michigan State University Extension and Sugarbeet Advancement, Frankenmuth, MI, United States; ^3^ Southern Minnesota Beet Sugar Cooperative, Renville, MN, United States; ^4^ Sugarbeet and Bean Research Unit, United States Department of Agriculture – Agricultural Research Services, East Lansing, MI, United States

**Keywords:** sugar beet (*Beta vulgaris* L.), *Beta vulgaris* (sugar beet), propane burner, leaf residue, integrated disease management, integrated pest management (IPM), tillage alternative, Mycosphaerellaceae

## Abstract

**Introduction:**

Sugarbeets account for 55 to 60% of U.S. sugar production. Cercospora leaf spot (CLS), primarily caused by the fungal pathogen *Cercospora beticola*, is a major foliar disease of sugarbeet. Since leaf tissue is a primary site of pathogen survival between growing seasons, this study evaluated management strategies to reduce this source of inoculum.

**Methods:**

Fall- and spring-applied treatments were evaluated over three years at two study sites. Treatments included standard plowing or tilling immediately post-harvest, as well as the following alternatives to tillage: a propane-fueled heat treatment either in the fall immediately pre-harvest or in the spring prior to planting, and a desiccant (saflufenacil) application seven days pre-harvest. After fall treatments, leaf samples were evaluated to determine *C. beticola* viability. The following season, inoculum pressure was measured by monitoring CLS severity in a susceptible beet variety planted into the same plots and by counting lesions on highly susceptible sentinel beets placed into the field at weekly intervals (fall treatments only).

**Results:**

No significant reductions in *C. beticola* survival or CLS were observed following fall-applied desiccant. The fall heat treatment, however, significantly reduced lesion sporulation (2019-20 and 2020-21, *P* < 0.0001; 2021-22, *P* < 0.05) and *C. beticola* isolation (2019-20, *P* < 0.05) in at-harvest samples. Fall heat treatments also significantly reduced detectable sporulation for up to 70- (2021-22, *P* < 0.01) or 90-days post-harvest (2020-21, *P* < 0.05). Reduced numbers of CLS lesions were observed on sentinel beets in heat-treated plots from May 26-June 2 (*P* < 0.05) and June 2-9 (*P* < 0.01) in 2019, as well as June 15-22 (*P* < 0.01) in 2020. Both fall- and spring-applied heat treatments also reduced the area under the disease progress curve for CLS assessed the season after treatments were applied (Michigan 2020 and 2021, *P* < 0.05; Minnesota 2019, *P* < 0.05; 2021, *P* < 0.0001).

**Discussion:**

Overall, heat treatments resulted in CLS reductions at levels comparable to standard tillage, with more consistent reductions across year and location. Based on these results, heat treatment of fresh or overwintered leaf tissue could be used as an integrated tillage-alternative practice to aid in CLS management.

## 1 Introduction

Sugarbeets are the source of approximately 20% of global sugar production (Rangel et al., 2020). The United States produces 36.75 million tons of sugarbeets annually from which 6.62 million tons of sugar is refined (USDA-NASS (United States Department of Agriculture, National Agricultural Statistics Services), 2019). Beet sugar comprises approximately 55-60% of total US sugar production (USDA (United States Department of Agriculture), 2021). In the U.S., Minnesota is the number one sugarbeet producer with 12 million tons grown annually and Michigan is the number four producer with 4-5 million tons grown annually (USDA-NASS (United States Department of Agriculture, National Agricultural Statistics Services), 2019). Cercospora leaf spot (CLS), caused by the fungal pathogen *Cercospora beticola* Sacc., is the most important foliar disease of sugarbeet in much of the world (Weiland and Koch, 2004; Jacobsen and Franc, 2009; Khan et al., 2009), including Minnesota and Michigan. The formation of CLS lesions decreases photosynthetic area which leads to sugar losses. Severe infection can lead to defoliation and further sugar losses when regrowth of leaves occurs (Franc, 2010). This disease can cause reduced root weight and sugar content, and yield losses of up to 50% may occur (Lamey et al., 1987; Shane and Teng, 1992). Fungicide management for CLS costs sugarbeet growers between $300-375 per hectare in Minnesota. In Michigan, severe CLS can cause an estimated $100 million in management costs and yield losses (*personal communication*, C. Guza, Michigan Sugar Company, 2022).

*Cercospora beticola* overwinters largely as pseudostromata present on infected leaf residue (Pool and McKay, 1916; McKay and Pool, 1918; Khan et al., 2008). Survival was shown to be reduced by burying leaf debris, which becomes more effective with increased depth and time. Inoculum on the soil surface survives anywhere from 20-22 months (Nagel, 1938; Khan et al., 2008) to two to three years (Pool and McKay, 1916; Solel, 1970), while inoculum buried at 10 to 20 cm decreased *C. beticola* survival to 10 months (Khan et al., 2008) or less (Solel, 1970). Pseudostromata in infected leaf debris are thought to be the primary inoculum source (Pool and McKay, 1916; Jacobsen and Franc, 2009) and dispersal of *C. beticola* can occur through the movement of infested plant material (Knight et al., 2018; Knight et al., 2019). Infected debris from weed hosts can also be an important inoculum source as it perpetuates CLS infection and inoculum in years when sugarbeet are not planted (Khan et al., 2008; Franc, 2010; Skaracis et al., 2010; Tedford et al., 2018; Knight et al., 2020). Further studies have identified potential alternative inoculum sources, such as infected seed (Knight et al., 2020; Spanner et al., 2022). Once *C. beticola* conidia are produced by the primary inoculum, they are dispersed by wind, water movement, and insects (McKay and Pool, 1918; Carlson, 1967; Lawrence and Meredith, 1970; Khan et al., 2007). Conidial dispersal distance can reach up to 100 m (McKay and Pool, 1918), which means it is important to consider neighboring sugarbeet fields during management.

Management of CLS relies on at least a three-year crop rotation with non-host crops, timely fungicide applications using disease prediction models, and the use of tolerant varieties (Windels et al., 1998; Khan et al., 2007; Jacobsen, 2010). None of these management strategies are effective on their own in areas with severe disease, and an integrated approach is necessary to keep CLS from causing economic damage. Host resistance is one of the primary management tools used against CLS. Unfortunately, there are no commercial varieties that have immunity to CLS (Smith and Gaskill, 1970; Smith and Ruppel, 1974; Rossi, 1999). While several newer varieties are highly tolerant (REACh (Michigan Sugarbeet Research and Education Advisory Council), 2020; REACh (Michigan Sugarbeet Research and Education Advisory Council), 2021), studies are ongoing to assess these varieties for root weight, sugar concentration, and various agronomic traits. As it is often difficult to maintain high recoverable sucrose yield in sugarbeet cultivars with high levels of diseases tolerance to CLS, development of tailored management programs to preserve desirable agronomic qualities are also required (Smith and Gaskill, 1970; Smith and Campbell, 1996).

While fungicides are extensively used for CLS management (Ruppel, 1986; Jacobsen and Franc, 2009), *C. beticola* is at high-risk for fungicide resistance development because of the numbers of fungicide applications each season (averages of 6-8 in severe epidemics in Michigan), high-level of genetic diversity in *C. beticola* populations (Vaghefi et al., 2016; Vaghefi et al., 2017a; Vaghefi et al., 2017b), and the numerous rounds of infection each season (McKay and Pool, 1918; Vereijssen et al., 2007). Reduced sensitivity to multiple fungicide groups including organotins, quinone outside inhibitors, demethylation inhibitors and benzimidazoles has been detected for *C. beticola* populations (Georgopoulos and Dovas, 1973; Ruppel and Scott, 1974; Giannopolitis, 1978; Cerato and Grassi, 1983; Bugbee, 1995; Karaoglanidis et al., 2000; Weiland and Halloin, 2001; Secor et al., 2010; Kirk et al., 2012; Rosenzweig et al., 2020). Thus, there is a critical need for integrated management strategies to control CLS in sugarbeet.

One understudied and underutilized strategy for managing CLS is the reduction of primary inoculum. In Michigan, evidence of infectious *C. beticola* spores has been found as early as April, with consistent early-season detections in 2017-2019 (Bublitz et al., 2021). Tedford et al. (2018) reported similar findings of airborne conidia present in early May in Ontario, Canada. Early detections of *C. beticola* spores from April through June in fields previously planted to beet support these as a primary inoculum source in North Central and Northeastern regions. This further provides evidence for successful overwintering of *C. beticola*, which may be present on leaf debris in the soil or on alternative hosts (Pool and McKay, 1916; Khan et al., 2008). In years between sugarbeet crops, infections of alternate hosts arising from overwintered sources would create a fresh source of inoculum in the field (Ruppel, 1986). Additionally, neighboring fields that were planted to beets the year prior will serve as an abundant source of inoculum.

Deep tillage has been shown to reduce *C. beticola* inoculum (Ruppel, 1986; Khan et al., 2008), however, this has become a less common practice in Michigan and Minnesota due to its disruption of soil structure. With the move to minimum tillage practices, alternative sanitation strategies are critical to enhance decomposition of leaves and potentially reduce inoculum. Studies of tillage-alternative residue management practices in apple, pear, and citrus have tested treatments such as urea, sugarbeet pulp, sugar cane pulp, dolomitic lime, fungal antagonists, and shredding of leaf litter (Spotts et al., 1997; Vincent et al., 2004; Heijne et al., 2006; van Bruggen et al., 2017). Similarly, herbicides used for preharvest defoliation or desiccation (Stahler, 1953) could accelerate leaf degradation with further potential to directly or indirectly impact disease (Altman and Campbell, 1977). Heating foliage to high temperatures could be further considered for potential sanitation due to the reports of 45.5°C being lethal to *C. beticola* (Pool and McKay, 1916). A propane-fueled foliar heat treatment impacted CLS lesion sporulation and viability of *C. beticola* for sugarbeets that were inoculated and grown in the greenhouse (Bublitz, 2019), supporting the potential for such a strategy. Strategic sanitation and inoculum management would reduce next-year disease pressure and have long-term economic, ecological, and environmental benefits.

This study aimed to i) assess potential end-of-season and early-season management strategies to reduce *C. beticola* inoculum levels and CLS severity in subsequent or current seasons and ii) investigate chemical and non-chemical sanitation practices as integrated tools to improve CLS management and reduce losses in root weight and sugar content. Treatments with the potential to reduce *C. beticola* overwintering and survival were tested in field experiments in Michigan and Minnesota. Three treatments were included in this study. One was a standard plow or tillage application to promote leaf degradation and reduce overwintering success in host material. The second treatment was a propane-fueled foliar heat treatment to directly reduce pathogen survival after exposure to high temperatures. The foliar heat treatment was applied in the spring to directly assess pathogen reduction (in Minnesota), or in the fall to incorporate potential increases in the rate of leaf degradation (in Michigan). The third treatment tested was a chemical desiccant to increase the rate of leaf degradation and reduce overwintering success in host material. To our knowledge, the current study is the first to test the use of in-field heat treatment and chemical desiccation for foliar disease management in sugarbeet.

## 2 Methods

### 2.1 Inoculum reduction trials of fall-applied treatments

#### 2.1.1 Trial information

The experiment was conducted at the Saginaw Valley Research and Extension Center (SVREC) in Frankenmuth, MI from 2019 to 2022 (**Table 1**). This location had Tappan-Londo loam soil with 0 to 3 percent slopes (USDA-NRCS (United States Department of Agriculture, Natural Resources Conservation Service), 2019) and the site received only natural precipitation. The trial consisted of a two-year experimental design to test treatments applied in the fall (at the end of the sugarbeet growing season) for the potential to reduce inoculum the following season. In all years, treatments were applied to four-row 3 m by 18 m plots, replicated four times, and arranged in a randomized complete block design (RCBD). Field tests of fall-applied treatments were repeated over two years.

**Table 1:** Field trial information for six studies on Cercospora leaf spot of sugarbeet conducted at the Saginaw Valley Research and Extension Center (SVREC) in Frankenmuth, MI and in Renville, MN from 2019-2022.

Location	#	Year	Planting date	Variety ^z,y^	Plot width x length (m)	Row spacing (cm)	Buffer spacing (m) ^x^	Harvest date	Inoculation date
SVREC 43.396543, -83.689057	1	2019	7-May	Crystal G333NT	3 x 18	76.2	3	24-Oct	9-Jul
		2020	17-Apr	Crystal G333NT	3 x 18	76.2	3	8-Oct	–
	2	2020	17-Apr	Crystal G932NT	3 x 18	76.2	3	16-Oct	23-Jul
		2021	7-May	Crystal G932NT	3 x 18	76.2	3	17-Sep	–
	3	2021	7-May	Crystal G932NT	3 x 18	76.2	3	3-Nov	12-Jul
		2022	29-Apr	Crystal G932NT	3 x 18	76.2	3	23-Sep	–
Renville 44.787496, -95.148788	1	2019	14-May	Beta 9230	3.4 x 3	55.9	1.5	–	–
	2	2020	12-May	Crystal RR018	3.4 x 3	55.9	1.5	–	–
	3	2021	12-May	Crystal M977	3.4 x 3	55.9	1.5	–	–

^z^ Cercospora leaf spot susceptible varieties selected based on Michigan Sugarbeet Research and Education Advisory Council variety trial results (REACh (Michigan Sugarbeet Research and Education Advisory Council), 2018; REACh (Michigan Sugarbeet Research and Education Advisory Council), 2019) and Southern Minnesota Beet Sugar Cooperative variety evaluations (SMBSC (Southern Minnesota Beet Sugar Cooperative), 2018; SMBSC (Southern Minnesota Beet Sugar Cooperative), 2019; SMBSC (Southern Minnesota Beet Sugar Cooperative), 2020)

^y^ Varieties were planted at rates of 123,500 seeds/ha at all SVREC trials and at 269,230 seeds/ha at all Renville trials. In Renville, the high seeding rate was to account for potential emergence issues in plots that were not tilled (heat and control).

^x^ SVREC trial buffers were planted to soybean (Trial 1 in 2019 and Trial 3 in 2021), corn (Trial 2 in 2020), or wheat (Trial 1 in 2020, Trial 2 in 2021, and Trial 3 in 2022). Renville trial buffers were planted to sugarbeets in all years.

#### 2.1.2 *Cercospora beticola* inoculation

In the first year of each two-year study, inoculations were made using a tractor-mounted field sprayer to apply a *C. beticola* spore solution (approximately 1x10^3^ spores/mL) at 140 L/ha. The conidial suspension was produced from dried CLS-symptomatic sugarbeet leaves collected the previous season, rehydrated and agitated in water, and filtered from leaf particulates (Eujayl et al., 2022). Symptomatic leaves were naturally- and artificially-infested with *C. beticola*, resulting in a representative mixture of local isolates. Inoculum was applied July 9, July 23, and July 12 in 2019, 2020, and 2021, respectively. Sugarbeets were grown to at least the 10-12 leaf growth stage prior to inoculation. Initial lesions were observed approximately 7-10 days after inoculation and severe CLS was typically observed by early-September each year, reaching a KWS (Kleinwanzlebener Saatzucht, 1970) CLS severity rating of 8-9 (on a 0-10 scale, see 2.5). To accurately assess impacts of treatments on overwintered inoculum from the first year, a susceptible sugarbeet variety was planted, with a 3-m buffer surrounding all plots (**Table 1**), and not inoculated or treated in the second year of each two-year study; the two-year sugarbeet rotation was used only as a research tool and does not represent recommended industry practices.

#### 2.1.3 Treatments

From 2019-20, the following fall treatments were evaluated: 1) non-treated control, 2) plow with a 3-m tandem disc set to invert soil 15-cm (6 inches) immediately post-harvest, 3) heat treatment using a custom designed 3.25-m wide propane-fueled, tractor-mounted shield burner initially designed for weed control (Multi-Trail Enterprises LLC; **Supplementary Figures 1A-D**) calibrated to heat foliage to 649-871°C at 1.6 kmph (1 mph) prior to defoliation, and 4) desiccant (saflufenacil; Sharpen 0.07 L/ha) applied seven days pre-harvest (McNaughton et al., 2015). The desiccant was applied with a CO_2_-powered backpack sprayer equipped with four 8004XR nozzles (76-cm spacing; TeeJet Technologies) calibrated at 140 L/ha. Methylated seed oil (1% v/v) surfactant and ammonium sulfate (2037 g/L) adjuvant were added to promote uptake and efficacy of the desiccant. The temperature of the heat treatment was measured prior to the study using several K thermocouple sensors connected to a S220-T8 data logger (Huato Electric Co., Ltd.). The thermocouple sensors were positioned at foliage level, at the soil surface level, and less than 1.25 cm beneath the soil surface as the burner was driven over them at 1.6 kmph. Beneath the burner implement, temperature notably decreased at and below ground levels (data not shown); thus, all heated plots were treated prior to defoliation to achieve high target temperatures at the canopy level. From 2020-21, experiments were repeated with the addition of the heat treatment applied at 4.8 kmph (3 mph). In 2021-22, the 4.8 kmph heat treatment was repeated for a second time with the non-treated control; as consistent performance was observed in 2019-20 and 2020-21 trials, the 1.6 kmph heat and desiccant treatments were not included.

### 2.2 Inoculum reduction trials of spring-applied treatments

#### 2.2.1 Trial information

Three experiments were conducted on a trial site near Renville, MN in 2019, 2020, and 2021 (**Table 1**). Soil types at this site ranged from Cordova-Rolfe complex clay to silt loams at 0 to 2 percent slopes to Normania loam at 1 to 3 percent slopes (USDA-NRCS, 2019). The season before treatment application, susceptible sugarbeets were grown and not treated with fungicide to achieve a KWS severity rating of 9 (at least 25% of leaf surface area impacted) by the end of September. Beets were defoliated in the fall and leaf residue was left on the soil surface to overwinter until spring. Treatments tested in this experiment were applied in the following spring the same day as planting. In all years, all treatments were applied to 3 m by 3.4 m plots, replicated four times, and arranged in an RCBD.

#### 2.2.2 Treatments

Three treatments similar to the fall applications were tested for inoculum reduction potential. Treatments included a 1) non-treated control, 2) tillage with a rotary tiller in the spring (prior to planting) to a depth of 10 cm to bury the residue and then raking by hand to create a firm seed bed for planting, and 3) propane burner application using a handheld Flame King Heavy Duty Propane Torch Weed Burner (Pico Rivera CA 90660; **Supplementary Figure 1E**) to the residue to target *C. beticola* survival over the winter. Sugarbeets were planted immediately after treatments were applied to the trial area.

### 2.3 Overwintering assessments for fall-applied treatments

#### 2.3.1 Symptomatic leaf samples

To evaluate survival of the pathogen over time, leaf samples from each treatment were assessed for percent lesion sporulation and isolation at 0-, 45-, 90-, and 135-days post-harvest (DPH) in the 2019-20 and 2020-21 trials. In 2021-22, leaf samples were assessed at 0-, 35-, 70-, and 168-DPH. All leaf samples contained eight sugarbeet leaves with distinct CLS lesions (between 0.1 to 3% symptomatic leaf area) that were arbitrarily collected from the middle canopy in the center two rows of each plot at harvest following treatment application. Post-harvest leaf samples were placed in mesh bags (approximately 66 cm by 37 cm, mesh size 5 mm^2^) and placed in the field. Bags were slightly incorporated (less than or equal to 2.5 cm) into the soil for all treatments except the plow treatment where the bags were buried 15.2 cm to simulate tillage effect on leaf residue.

#### 2.3.2 Leaf degradation

In 2019-20 and 2020-21, at-harvest leaf samples were stored in a cold room at 4°C for four days prior to destructive sampling. After collection from the field, post-harvest leaf samples were stored for three days at 4°C, rinsed with tap water over a 2.00-mm sieve to remove soil debris, and then left to air dry overnight between paper towels, for a total of four days after collection. In 2021-22, at-harvest leaf samples were stored at 4°C for five days prior to destructive sampling; post-harvest leaf samples were stored for four days at 4°C then rinsed and dried as described, for a total of five days after collection. At-harvest samples were collected directly from sugarbeet plants prior to defoliation and did not require rinsing to remove soil residue.

In 2019, leaf samples were photographed four days after collection and later measured using ImageJ software (Schneider et al., 2012). The total leaf area was calculated for each replicate sample and then divided by the number of leaves in each sample to estimate the standardized leaf area for each plot. To more accurately measure leaf degradation in 2020 and 2021, leaf samples were weighed at harvest and four or five days after recovery from the field, respectively. Percent leaf degradation was calculated by subtracting final weight from initial weight and dividing by the initial weight at harvest.

#### 2.3.3 *Cercospora beticola* viability: lesion sporulation

After collection and handling as described, all eight leaves from each sample were placed in moist chambers at room temperature (21-24°C) with ambient light conditions to induce sporulation of CLS lesions (Bublitz, 2019). Moist chambers were composed of 3.8-liter plastic resealable bags with a moist paper towel wetted with deionized water. Bags were inflated gently with ambient air. After three days, percent CLS lesion sporulation was measured by counting the number of sporulating lesions on each leaf using a dissecting binocular stereo microscope (7-10x magnification; Leica ZOOM 2000) and dividing by the total number of lesions assessed on each leaf.

#### 2.3.4 *Cercospora beticola* viability: lesion isolation

Ten lesions were arbitrarily selected from across each sample of eight leaves and excised using a 5-mm diameter cork-borer. The lesions were surface disinfested using an 8.25% sodium hypochlorite solution for 30 seconds, triple rinsed with sterile deionized water, air-dried on a sterile paper, and placed on 1.5% water or rose bengal agar (Millipore Sigma) with 0.5 g/L streptomycin and 0.25 g/L ampicillin to inhibit bacterial growth. Hyphal tip transfers (Brown, 1924) onto clarified V8 (CV8) agar (Miller, 1955) media amended with CaCO_3_ (0.8 g/L) with 0.5 g/L streptomycin and 0.25 g/L ampicillin were used to isolate pure cultures of *C. beticola* based on morphological characteristics. Hyphal tip transfers were done twice to ensure pure cultures of *C. beticola* (Forsyth et al., 1963). Percent CLS lesion viability was calculated by dividing the number of successful *C. beticola* isolations by the total number of lesions plated.

### 2.4 Early-season sentinel beet sampling for fall-applied treatments

#### 2.4.1 Live spore trap maintenance and in-field exposure

In 2020, 2021, and 2022, highly CLS susceptible beets (germplasm F1042) were used to assess the efficacy of inoculum reduction strategies (USDA-ARS (United States Department of Agriculture, Agricultural Research Service), 2017). These highly susceptible sugarbeets were referred to as “sentinel beets” because they were used to monitor in-field spore presence (Bublitz et al., 2021). In the MSU Plant Science Research Greenhouse Complex, F1042 sugarbeet seeds were mass planted in SureMix growing media (Michigan Grower Products, Inc.). Four sugarbeet seedlings at the cotyledon growth stage were transplanted to each planting box (61.0 cm by 30.5 cm) and maintained in the same greenhouse. Plants were fertilized at planting with Osmocote Smart-Release Plant Food Flower & Vegetable (Scotts Miracle-Gro, N-P-K 14-14-14) and approximately once per month with Osmocote Smart-Release Plant Food Plus Outdoor & Indoor (Scotts Miracle-Gro, N-P-K 15-9-12 plus micro- and secondary nutrients) according to labeled rates. Plants also were watered two to three times a week depending on greenhouse conditions. Nontarget diseases and insect pests impacting sentinel beets, e.g., powdery mildew, thrips, and aphids, were monitored and managed by greenhouse staff; to reduce potential residual effects on *C. beticola*, Sulfur Plant Fungicide (sulfur 90%) was applied as necessary to manage powdery mildew (2-3 applications per year).

Once the sugarbeets reached the 10-14 leaf stage, one box of sentinel beets was placed in three of the four replicate field plots for each treatment. Boxes were left in the field for seven days before returning to the greenhouse, starting at 7 to 39 days after planting (DAP) of the field experiments and continuing until 60 to 95 DAP (**Supplementary Table 1**). Sentinel beets were used as live spore traps to estimate early levels of viable inoculum in each plot. Of note, an insecticide treatment of Mainspring (cyantraniliprole 18.66%) at 236.6 ml/3.8 L (8 fl oz/100 gal) was applied to sentinel beets seven days prior to field exposure to reduce leaf miner and thrips infestation in the field. While in the field, wire cages (constructed from poultry netting) were secured onto the planting box to reduce animal feeding and disturbance. Sentinel plants were also manually watered twice weekly dependent on occurrence of natural rain events.

#### 2.4.2 Live spore trap incubation and symptom observation

After seven days of exposure in the field, sentinel beets (one box of four beets collected from each of three replicate plots per treatment) were placed in a humidity chamber to provide favorable conditions for *C. beticola* infection. The chamber consisted of steel shelves lined and enclosed with plastic sheeting (4-mm thick clear poly) and was kept at a temperature of 27°C with humidifiers supplying a humidity greater than 95% (Bublitz et al., 2021). After three days in the humidity chamber, the boxes of beets were returned to the greenhouse for three weeks. Greenhouse temperatures varied between 20 to 30°C degrees and received natural light (Bublitz et al., 2021). Characteristic CLS lesions (Windels et al., 1998; Weiland and Koch, 2004) were identified and counted on each plant and the total number of lesions for each box of beets was recorded. Lesions were only counted if pseudostromata, a distinguishing characteristic of Cercospora leaf spots, were detected using a hand lens (3x to 6x magnification).

A non-inoculated control was incubated with field-exposed sentinel beets to monitor secondary dispersal of conidia within the humidity chamber. Asymptomatic sentinel beets of the same age as the field-exposed beets were kept separately in the greenhouse before placing in the moist chamber. No to low (1-6 lesions) CLS symptoms were observed on the non-inoculated controls during the study.

### 2.5 Disease pressure assessments for fall- and spring-applied treatments

Disease severity data were collected from the middle two rows of each plot. The KWS (Kleinwanzlebener Saatzucht, 1970) CLS standard surface area rating scale was used for fall-applied treatment evaluations in 2020 and 2021 and all spring-applied treatment evaluations. The KWS scale ranges from 1 to 10, in which 1 = 1-5 spots/leaf (0.1% severity), 2 = 6-12 spots/leaf (0.35% severity), 3 = 13-25 spots/leaf (0.75% severity), 4 = 26-50 spots/leaf (1.5% severity), 5 = 51-75 spots/leaf (2.5% severity), 6 = 3% severity (proven economic damage), 7 = 6% severity, 8 = 12% severity, 9 = 25% severity, and 10 = 50% to 100% severity.

To more easily evaluate whole plant symptoms, the Agronomica (Battilani et al., 1990) CLS severity scale (0-5) was used in 2022 and standardized to a 0-10 scale, where 0 = healthy foliage, 1 = a single isolated spot on some leaves, 2 = 50% of outer leaves show one to a few spots ~20 per leaf, 3 = outer leaves ~50% foliage show 20-100 spots per leaf, 4 = nearly all outer leave are affected by several spots (still isolated), 5 = some (2-4) outer leaves show coalescence of spots to necrotic areas (first spots appear on the inner leaves), 6 = fully and almost grown leaves show several coalesced necrotic areas of 1-2 cm in diameter (that do not lead to large necrotic areas), 7 = some (2-4) outer leaves show relatively large necrotic areas (20-30% of the leaf area), 8 = for the first time some leaves (2-8) show 80-100% severity, 9 = the entire foliage is strongly affected, 10 = the original foliage is completely destroyed.

Disease observations were collected biweekly from mid-June until harvest in late-August or early-September. Area under the disease progress curves were calculated using CLS severity ratings in the below equation:


$$A_{k}=\sum_{i=1}^{N_{i}-1}\frac{\left(y_{i}+y_{i+1}\right)}{2}(t_{i+1}-t_{i})$$

The rating time points in a sequence are considered (t_i_) and the associated measures of the disease level (y_i_); y(0) = y_0_ is defined as the initial infection or the disease level at t = 0. *A*(*t_k_*), the AUDPC at *t = t_k_*, is the total accumulated disease until *t = t_k_* (Madden et al., 2017).

### 2.6 Yield and sugar assessments for fall-applied treatments

In the second year of each trial, two center rows of each treatment plot were mechanically harvested and weighed to determine yield. Root subsamples (approximately 10 kg) were collected from the center two rows of each plot. Sugar analysis was conducted by Michigan Sugar Company (Bay City, MI) as described in Tedford et al. (2019) to assess percent sugar and recoverable white sugar per ton (RWST). Samples were sliced using a rasping circular saw to obtain 1 L of root pulp (brei), which was filtered and the juice extracted for sucrose yield and standard quality analysis (Carruthers & Oldfield, 1961). The polarimeter method was used to determine sucrose content (Halvorson et al., 1978). Methods reported by Last et al. (1976) were used to determine sucrose purity (clear juice purity, CJP) and brei impurity amino-N. Recoverable white sucrose per metric ton of fresh beets (RWS) was calculated as in Van Eerd et al. (2012) and converted to recoverable white sucrose per hectare (RWSH) using the following equation: RWSH (metric ton/hectare) = RWS (kg/metric ton) × Total Yield (metric ton/hectare) ÷ 1000. As disease impacts were the primary focus in studies evaluating spring-applied treatments, yield and sugar were not measured.

### 2.7 Statistical analyses

For all experiments, treatment was evaluated as the fixed effect of interest and replicate was considered a random effect. Due to differences in experimental treatments and design, years and locations were analyzed separately. Analysis of variance (ANOVA) was conducted using SAS (Statistical Analysis System) v. 9.4 software package (SAS Institute, Inc. Cary, NC, United States) to determine treatment effects on percent *C. beticola* sporulation and isolation, standardized leaf area, percent leaf degradation, early-season lesion counts from sentinel beets, AUDPC, yield, percent sugar, RWS, and RWSH values. Sentinel beet lesion count data were normalized using the lognormal distribution option to best fit this data (Gbur et al., 2012). Statistical analyses (mixed model ANOVA) were conducted using the generalized linear mixed model (GLIMMIX) procedure (SAS Institute Inc., 2013) and evaluated at the α = 0.05 significance level. Fisher’s protected least significance difference (LSD) was used for mean comparisons. LSD was calculated to compare treatment differences using letter separation option “mult” macro (Piepho, 2012).

## 3 Results

### 3.1 Leaf degradation after fall-applied treatments

No differences were detected in standardized leaf area for treatments tested 2019-20 (*P* = 0.0757; **Table 2**). However, significant differences in percent leaf degradation were detected in 90- (*P*< 0.05) and 135-DPH (*P*< 0.01) samples for treatments tested 2020-21, with observable differences in leaf tissue recovered (**Supplementary Figure 2**).

**Table 2:** Standardized leaf area and percentage sugarbeet leaf degradation for at-harvest and soil incorporated post-harvest samples^z,y^ collected 0-, 45-, 90-, and 135-days post-harvest (DPH) from fall-applied field studies in 2019-2020 and 2020-2021.

Treatment^z^	2019-2020				2020-2021					
	Standardized Leaf Area (cm^2^) ^x^				Leaf Degradation (%) ^w,v^					
	0-DPH	45-DPH	90-DPH	135-DPH	0-DPH	45-DPH	90-DPH		135-DPH	
Control	43.8	58.6	11.8	53.1	12.9	58.5	72.6	bc	80.4	bc
Plow	44.6	51.8	19.7	50.3	19.1	62.4	82.6	a	86.2	ab
Heat (1.6 kmph)	33.2	42.8	19.0	28.1	7.8	63.6	81.9	ab	89.3	a
Desiccant	38.7	55.1	14.5	48.8	14.1	53.9	67.7	c	74.3	c
Heat (4.8 kmph)	–	–	–	–	20.3	68.2	71.1	c	86.2	ab
*SE*	*2.6*	*6.5*	*5.8*	*8.2*	*2.8*	*3.4*	*3.1*		*2.5*	
*P-value* ^u^	*0.0757*	*0.3600*	*0.7275*	*0.2727*	*0.0625*	*0.0654*	0.0177 *		0.0014 **	
*LSD*	*-*	*-*	*-*	*-*	*-*	*-*	*9.6*		*6.2*	

^z^ Non-treated control, plow with a 3-m tandem disc set to invert soil 15 cm. immediately post-harvest, heat treatment using a propane-fueled burner (Multi-Trail Enterprises LLC) calibrated to heat foliage to 649-871°C at 1.6 kmph and 4.8 kmph prior to defoliation, and a desiccant (Sharpen 0.07 L/ha, methylated seed oil 1% v/v, ammonium sulfate 2037 g/L) applied seven days pre-harvest.

^y^ Measurements were collected from independent sets of leaves at each timepoint, not repeated measurements from the same sets of leaves over time.

^x^ Average of the total standardized leaf area (total area divided by number of leaves) quantified using ImageJ (Schneider et al., 2012).

^w^ Percent leaf degradation calculated using initial leaf weights at-harvest and final weights post-harvest [(Initial – Final)/Initial].

^v^ Column values followed by the same letter were not significantly different based on Fisher’s Protected LSD (α = 0.05).

^u^ Asterisk designations correspond to p-value thresholds<0.05 *,<0.01 **.

In 2020-21, heat treatment at 4.8 kmph and desiccant treatments resulted in leaf degradation comparable to the control at all timepoints. Leaf degradation resulting from the plow treatment was significantly greater than degradation in the control at 90-DPH (*P*< 0.05) and was comparable to the 1.6-kmph heat treatment at 90- and 135-DPH. However, differences between the plow and control treatments were not detectable by 135-DPH. In the repeated test conducted in 2021-22, the heat treatment at 4.8 kmph resulted in significantly reduced leaf degradation compared to the control at 0-DPH (**Table 3**) but was comparable to the control at all subsequent timepoints.

**Table 3:** Percent sugarbeet leaf degradation for at-harvest and soil-incorporated post-harvest samples^z,y^ collected 0-, 35-, 70-, and 168-days post-harvest (DPH) from fall-applied treatments in field studies in 2021-2022.

Treatment ^z^	2021-2022				
	Leaf Degradation (%) ^x,w^				
	0-DPH		35-DPH	70-DPH	168-DPH
Control	20.1	a	40.9	75.1	91.4
Heat (4.8 kmph)	12.9	b	37.1	63.7	90.9
*SE*	*1.7*		*7.8*	*5.5*	*1.0*
*P-value* ^v^	0.0211 *		*0.2731*	*0.0691*	*0.7468*
*LSD*	*5.2*		*-*	*-*	*-*

^z^ Non-treated control and heat treatment using a propane-fueled burner (Multi-Trail Enterprises LLC) calibrated to heat foliage to 649-871°C 4.8 kmph prior to defoliation.

^y^ Measurements were collected from independent sets of leaves at each timepoint, not repeated measurements from the same sets of leaves over time.

^x^ Percent leaf degradation calculated using initial leaf weights at-harvest and final weights post-harvest [(Initial – Final)/Initial].

^w^ Column values followed by the same letter were not significantly different based on Fisher’s Protected LSD (α = 0.05).

^v^ Asterisk designations correspond to p-value thresholds<0.05 *.

### 3.2 *Cercospora beticola* survival after fall-applied treatments

In the 2019-20 trial, significant treatment differences were detected in percentage of CLS lesion sporulation (*P*< 0.0001) and *C. beticola* isolation frequencies (*P*< 0.05) for 0-DPH samples (**Table 4**). Compared to the non-treated control, CLS lesion sporulation and *C. beticola* isolation were reduced in leaf samples from heat-treated plots by 99 and 91%, respectively. No significant differences were detected in sporulation or isolation frequencies of *C. beticola* from leaf samples evaluated at 45-, 90-, or 135-DPH in this trial (*P* > 0.05).

**Table 4:** Cercospora leaf spot lesion sporulation (Sp) and *C. beticola* isolation frequencies (Is) from soil-incorporated sugarbeet leaf samples collected from Michigan studies at post-harvest timepoints following fall-applied treatments in 2019-2020, 2020-2021, and 2021-2022.

Trial Year	Treatment ^z^	0-DPH ^y^				45-DPH			90-DPH			135-DPH	
		Sp ^x,w^ (%)		Is ^v^ (%)		Sp (%)		Is (%)	Sp (%)		Is (%)	Sp ^u^ (%)	Is (%)
2019-20	Control	78.1	a	38.3	a	17.6		0.0	1.2		0.0	0.3	0.0
	Plow	60.3	b	43.3	a	0.6		0.0	0.0		0.0	0.0	0.0
	Heat (1.6 kmph)	1.1	c	3.3	b	0.7		0.0	0.0		0.0	0.0	0.0
	Desiccant	59.8	b	38.3	a	13.2		0.0	0.6		0.0	0.1	0.0
	*SE*	*4.5*		*10.3*		*7.4*		*0.0*	*0.5*		*0.0*	*0.1*	*0.0*
	*P-value* ^t^	< 0.0001 ***		0.0235 *		*0.2601*		*NS*	*0.1879*		*NS*	*0.158*	*NS*
	*LSD*	*13.4*		*25.9*		*-*		*-*	*-*		*-*	*-*	*-*
2020-21	Control	77.9	a	5.0		22.2	a	0.0	7.2	a	2.5	8.8	0.0
	Plow	66.4	ab	2.5		0.0	b	0.0	0.0	b	0.0	1.6	0.0
	Heat (1.6 kmph)	0.0	c	0.0		0.0	b	0.0	0.0	b	0.0	0.0	0.0
	Desiccant	50.1	b	7.5		32.2	a	0.0	7.5	a	0.0	0.9	0.0
	Heat (4.8 kmph)	7.7	c	0.0		0.0	b	0.0	0.3	b	0.0	0.3	0.0
	*SE*	*5.8*		*3.3*		*6.2*		*0.0*	*1.9*		*1.1*	*2.7*	*0.0*
	*P-value*	< 0.0001 ***		*0.4802*		0.0083 **		*NS*	0.0267 *		*0.4449*	*0.2113*	*NS*
	*LSD*	*19.7*		*-*		*19.8*		*-*	*6.1*		*-*	*-*	*-*
		0-DPH				35-DPH			70-DPH			168-DPH	
2021-22	Control	90.8	a	7.5		10.1	a	0.0	2.9	a	0.0	0.0	0.0
	Heat (4.8 kmph)	62.8	b	10.0		0.6	b	7.5	0.0	b	0.0	0.0	0.0
	*SE*	*4.3*		*4.4*		*1.3*		*3.4*	*0.2*		*0.0*	*0.0*	*0.0*
	*P-value*	0.0183 *		*0.3910*		0.0202 *		*0.2152*	0.0022 **		*NS*	*NS*	*NS*
	*LSD*	*19.0*		*-*		*6.6*		*-*	*0.9*		*-*	*-*	*-*

^z^ Non-treated control, plow with a 3-m tandem disc set to invert soil 15 cm. immediately post-harvest, heat treatment using a propane-fueled burner (Multi-Trail Enterprises LLC) calibrated to heat foliage to 649-871°C at 1.6 kmph and 4.8 kmph prior to defoliation, and a desiccant (Sharpen 0.07 L/ha, methylated seed oil 1% v/v, ammonium sulfate 2037 g/L) applied seven days pre-harvest.

^y^ Days post-harvest (DPH).

^x^ Percent lesion sporulation (Sp) determined following a 3-d incubation in a moist chamber at 21-23.9°C. Lesion sporulation assessed for 1,636 to 3,895 lesions per timepoint (across all treatments) in 2019. Lesion sporulation assessed for 1,020 to 1,600 lesions per timepoint in 2020. Lesion sporulation assessed for 312 to 548 lesions per timepoint in 2021.

^w^ Column values followed by the same letter were not significantly different based on Fisher’s Protected LSD (α = 0.05).

^v^ Frequency of *C. beticola* isolation (Is) determined from morphological confirmation of *C. beticola* growth from 15 (2019) and 10 (2020 and 2021) representative lesions, plated on half-strength clarified V8 juice agar (Miller, 1955) amended with 0.5 g/L streptomycin and 0.25 g/L ampicillin.

^u^ Late-winter sporulation observations may be limited by unknown lesion maturity (e.g., number of prior in-season sporulation events).

^t^ Asterisk designations correspond to p-value thresholds<0.05 *,<0.01 **,<0.001 ***; NS indicates no significant differences were detected as data were all zeroes.

In the 2020-21 trial, significant reductions in percent lesion sporulation were observed at 0-DPH (*P*< 0.0001), 45-DPH (*P*< 0.01), and 90-DPH (*P*< 0.05) for leaf samples in heat treated plots compared to the control (**Table 4**). Percentage point reductions in sporulation were greater than 70% at-harvest, 20% at 45-DPH, and 5% at 90-DPH for 1.6- and 4.8-kmph heat treatments compared to the control. Percent sporulation was reduced to 0% for the 1.6-kmph heat treatment at each time point and 0.3% or lower for the 4.8-kmph treatment. No significant differences were observed in lesion sporulation for samples evaluated at 135-DPH (*P* > 0.05). No differences were detected in isolation frequencies of *C. beticola* from leaf samples evaluated at 0-, 45-, 90-, or 135-DPH (*P* > 0.05).

In the 2021-22 trial, the 4.8-kmph treatment significantly reduced percent sporulation for at-harvest, 35-, and 70-DPH samples compared to the control (*P<* 0.05, **Table 4**). A 31% decrease in percent sporulation was seen for the heat-treated samples compared to the control at-harvest. There were no significant differences between percent sporulation for the 4.8-kmph heat treated and control samples at 168-DPH or percent isolation at any sampling time.

In both the 2019-20 and 2020-21 trials, lesion sporulation was significantly reduced in the desiccant treatment compared to the control at 0-DPH (**Table 4**). This reduction, however, was not consistent at remaining overwintering timepoints in either year. The plow treatment did not result in consistent reductions of lesion sporulation in 2019-20 samples but significantly reduced lesion sporulation in 2020-21 samples collected 45- and 90-DPH.

### 3.3 Early-season inoculum assessments after fall-applied treatments

In 2020, following 2019 fall-applied treatments, reduced numbers of CLS lesions were observed on live spore traps (sentinel beets) in heat-treated plots from May 26-June 2 (*P*< 0.05) and June 2-9 (*P*< 0.01) (**Table 5**), up to 46 to 53 days after planting. The number of CLS lesions observed on sentinel beets in the plow and desiccant treatments were not significantly different from the control at any sampling timepoint.

**Table 5:** Number of Cercospora leaf spot lesions observed on live spore traps (sentinel beets) placed in Michigan field studies in 2020, 2021, and 2022 (the year following fall-applied treatments).

Trial Year	Treatment ^z^	Sentinel ^y,x^							
		*May 26 – June 2* ^w^			*June 2 – June 9*			*July 14 – July 21*	
2019-20	Control	5.5	(284)	a	4.1	(60)	b	5.3	(199)
	Plow	5.4	(337)	a	3.9	(51)	b	5.4	(219)
	Heat (1.6 kmph)	4.0	(65)	b	2.5	(16)	c	4.7	(130)
	Desiccant	6.1	(482)	a	5.3	(212)	a	5.7	(320)
	*SE*	*0.4*			*0.3*			*0.2*	
	*P-values* ^v^	*0.0323*	*		*0.0063*	**		*0.0653*	
	*LSD*	*1.3*			*1.1*			*-*	
		*June 1 – June 8*			*June 15 – June 22*			*June 29 – July 6*	
2020-21	Control	2.9	(19)		6.7	(888)	ab	7.3	(1514)
	Plow	2.0	(9)		6.3	(628)	ab	7.2	(1397)
	Heat (1.6 kmph)	1.2	(4)		4.9	(144)	c	7.0	(1189)
	Desiccant	2.1	(15)		7.0	(1239)	a	7.1	(1222)
	Heat (4.8 kmph)	0.5	(2)		5.9	(396)	b	7.3	(1498)
	*SE*	*0.7*			*0.3*			*0.2*	
	*P-values*	*0.1128*			*0.0027*	**		*0.8198*	
	*LSD*	*-*			*0.8*			*-*	
		*May 17 – May 24*			*May 24 – May 31*			*June 15 – June 22*	
2021-22	Control	5.1	(166)		6.4	(614)		5.7	(358)
	Heat (4.8 kmph)	4.3	(98)		5.6	(305)		5.8	(391)
	*SE*	*0.4*			*0.2*			*0.4*	
	*P-value*	*0.1926*			*0.2238*			*0.7912*	
	*LSD*	*-*			*-*			*-*	

^z^ Non-treated control, plow with a 3-m tandem disc set to invert soil 15 cm. immediately post-harvest, heat treatment using a propane-fueled burner (Multi-Trail Enterprises LLC) calibrated to heat foliage to 649-871°C at 1.6 kmph and 4.8 kmph prior to defoliation, and a desiccant (Sharpen 0.07 L/ha, methylated seed oil 1% v/v, ammonium sulfate 2037 g/L) applied seven days pre-harvest.

^y^ Means generated under the lognormal distribution option in the GLIMMIX procedure (SAS v 9.4) of total CLS lesions counted on sentinel beets (USDA germplasm F1042) after 1-week exposure in the field, 3 d incubation in a 25°C humidity chamber, and 2 weeks in a greenhouse. (Non-normally distributed mean estimates shown in parentheses).

^x^ Column values followed by the same letter were not significantly different based on Fisher’s Protected LSD (α = 0.05).

^w^ Data shown for sampling weeks: 1^st^, 2^nd^, and 7^th^ (2020); 2^nd^, 3^rd^, and 4^th^ (2021); 1^st^, 2^nd^, 4^th^ (2022). No or low detections in weeks not shown due to low spore concentrations or other external insect or drought stress factors (**Supplementary Table 1**).

^v^ Asterisk designations correspond to p-value thresholds<0.05 *,<0.01**.

In 2021, following 2020 fall-applied treatments, the 1.6-kmph heat treatment resulted in significantly fewer lesions on sentinel beets sampled June 15-22 (*P*< 0.005, **Table 5**), 39 to 46 days after planting. No significant differences were seen in treatments sampled June 1-8 (*P =* 0.1128). Inoculum levels in the plow and desiccant treatments were again not significantly different from the control at any sampling timepoint.

In 2022, following 2021 fall-applied treatments, no significant differences were detected between the number of lesions for the 4.8-kmph heat treatment compared to the control (**Table 5**).

### 3.4 Early development of CLS after fall-applied and spring-applied treatments

**Figure 1:**
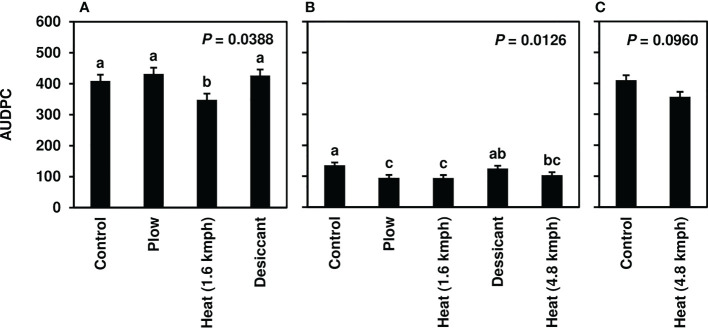
Mean area under the disease progress curve (AUDPC) values from Michigan field studies collected in **(A)** 2020, **(B)** 2021, and **(C)** 2022, the years following fall-applied treatments. Treatments included were a non-treated control, plow with a 3-m tandem disc set to invert soil 15 cm. immediately post-harvest, heat treatment using a propane-fueled burner (Multi-Trail Enterprises LLC) calibrated to heat foliage to 649-871°C at 1.6 kmph and 4.8 kmph prior to defoliation and a desiccant (Sharpen 0.07 L/ha, methylated seed oil 1% v/v, ammonium sulfate 2037 g/L) applied seven days pre-harvest. AUDPC values were calculated according to Madden et al. (2017) using severity ratings collected at 5 timepoints; ratings were based on the KWS severity scale (0-10) in 2020 and 2021 and the Agronomica severity scale (standardized to 0-10) in 2022. Bars represent the means of four replicate plots and error bars represent standard errors. Bars with the same letter were not significantly different based on Fisher’s Protected LSD (α = 0.05). Analyses were conducted within each year.

**Figure 2:**
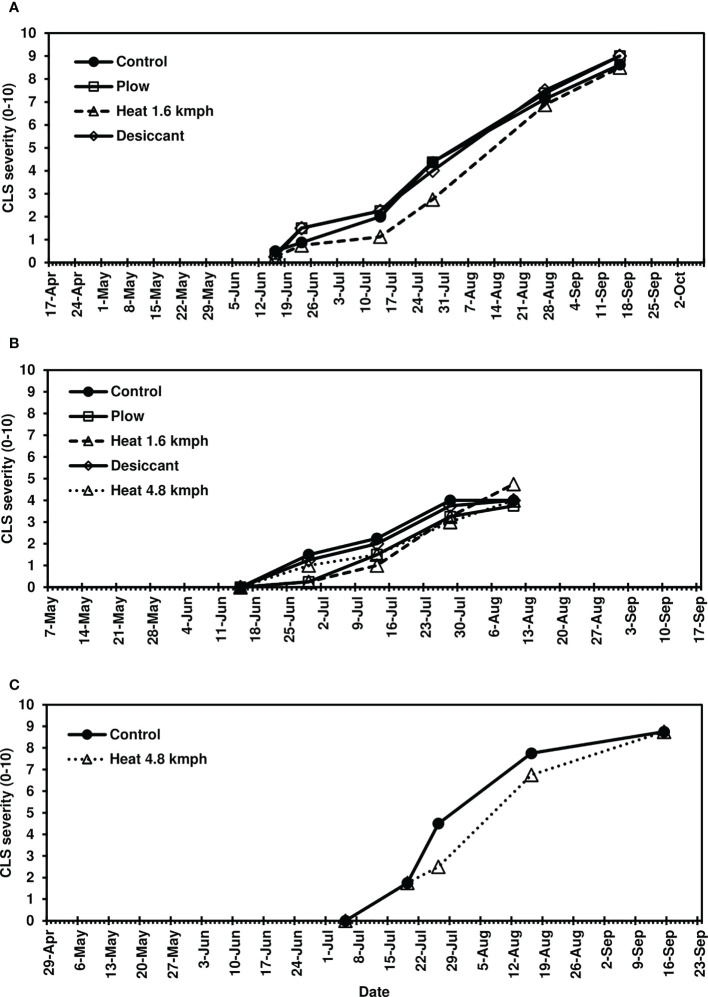
Mean Cercospora leaf spot severity progression on sugarbeet in **(A)** 2020, **(B)** 2021, and **(C)** 2022, following fall-applied treatments evaluated in Frankenmuth, MI. Treatments included a non-treated control, plow with a 3-m tandem disc set to invert soil 15 cm. immediately post-harvest, heat treatment using a propane-fueled burner (Multi-Trail Enterprises LLC) calibrated to heat foliage to 649-871°C at 1.6 kmph and 4.8 kmph prior to defoliation, and a desiccant (Sharpen 0.07 L/ha, methylated seed oil 1% v/v, ammonium sulfate 2037 g/L) applied seven days pre-harvest. In 2020 and 2021, CLS ratings were based on the KWS severity scale (0-10) in 2020 and 2021 where 0 is 0.1% severity (1-5 spots per leaf) and 10 is 50% severity. In 2022, the CLS ratings were based on the Agronomica (Battilani et al., 1990) severity scale standardized to 0-10 scale where 0 is completely healthy foliage and 10 is completely destroyed original foliage with respect to CLS. Each point represents a mean of four replicate field plots. Date axes start at the planting date and end at the harvest date for each year.

Following fall-applied heat treatments, next-year CLS was monitored until moderate to severe disease severity levels were achieved in 2020 (8-10), 2021 (3-6), and 2022 (8-10). No fungicide treatments were applied to this trial, and disease naturally progressed beyond these assessments until high levels of CLS developed across all treatments. Analyses focused, therefore, on early-season disease development 39-139 days after planting. The fall heat treatment significantly reduced CLS pressure in 2020 and 2021 (*P*< 0.05) compared to the non-treated control (**Figures 1A, B**). Greater than 35% reductions in AUDPC were observed in fall heat-treated plots at 1.6 kmph in 2020. In 2021, greater than 25, 30, and 20% reductions were measured for the plow, 1.6-kmph, and 4.8-kmph heat treatment, respectively. Upon further investigation, following 1.6-kmph heat treatment visual reductions in CLS severity were sustained for six to eight weeks of the growing season (**Figures 2A, B**). No significant differences between treatment AUDPC were observed in 2022 (**Figure 1C**). CLS severities following the 4.8-kmph heat treatment, however, were reduced for approximately four weeks of the growing season (**Figure 2C**).

**Figure 3:**
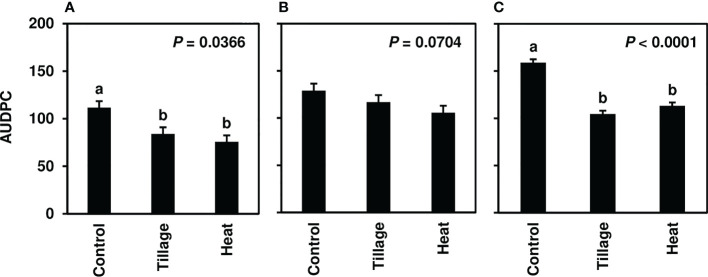
Mean area under the disease progress curve (AUDPC) values from Minnesota field studies in **(A)** 2019, **(B)** 2020, and **(C)** 2021. Treatments included a non-treated control, tillage with a rotary tiller in the spring (prior to planting) to a depth of 10 cm to bury the residue, and propane burner heat application using a handheld Flame King Heavy Duty Propane Torch Weed Burner (Pico Rivera CA 90660). AUDPC values were calculated according to Madden et al. (2017) using severity ratings collected at 8-9 timepoints; ratings represented scores assigned by two to four raters and were based on the KWS severity scale (0-10). Bars represent the means of four replicate plots and error bars represent standard errors. Bars with the same letter were not significantly different based on Fisher’s Protected LSD (α = 0.05). Analyses were conducted within each year.

**Figure 4:**
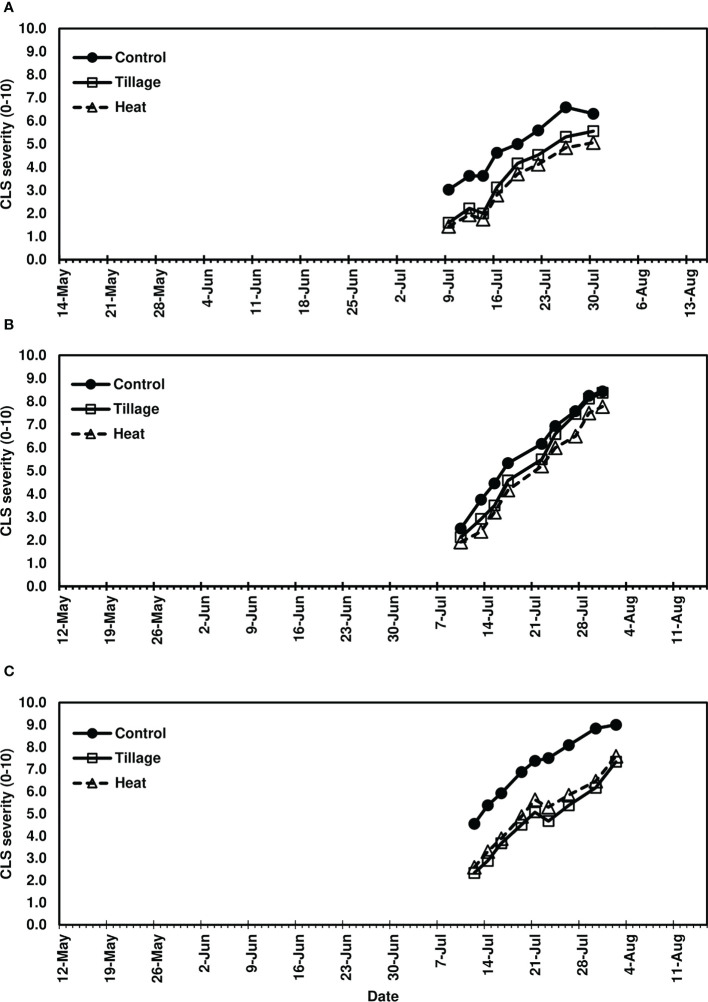
Mean Cercospora leaf spot severity progression in **(A)** 2019, **(B)** 2020, and **(C)** 2021, following spring-applied treatments evaluated in Renville, MN. Treatments included a non-treated control, tillage with a rotary tiller in the spring (prior to planting) to a depth of 10 cm to bury the residue, and heat treatment of residue using a propane-fueled using a handheld Flame King Heavy Duty Propane Torch Weed Burner (Pico Rivera CA 90660) immediately prior to planting. CLS ratings were based on the KWS severity scale (0-10) where 0 is 0.1% severity (1-5 spots per leaf) and 10 is 50% severity. Each point represents a mean of four replicate field plots. Date axes start at the planting date and end August 15 for each year as trials were not harvested.

In studies of spring-applied heat treatments, same-year disease development was assessed until moderate to severe CLS levels were achieved in 2019 (4-8.5), 2020 (7-9), and 2021 (7-9). AUDPC was significantly lower for plots following spring (pre-plant) tillage and heat treatment application of infected leaf residue in 2019 (*P*< 0.05) and 2021 (*P*< 0.0001) (**Figures 3A, C**) but not in 2020 (*P* = 0.0704; **Figure 3B**); significant reductions corresponded to three to four weeks of visual CLS severities less than control plots (**Figures 4A, C**). In 2020, visible differences in CLS severities were detected for about four weeks of the growing season, though less delineation between treatments was observed than in other years (**Figure 4B**).

### 3.5 Final yield and sugar after fall-applied treatments

No treatments yielded significantly differently from the control in any year (*P* > 0.05) (**Table 6**). There were no statistical differences detected in percent sugar, RWS, or RWSH for any treatments in the 2019-20, 2020-21, or 2021-22 trials. Overall, the heat treatment did not have any beneficial or deleterious effects on sugarbeet root or sugar yield at- or post-harvest (data not shown).

**Table 6:** Sugarbeet yield, percent sugar content, and recoverable white sugar from Michigan field studies collected in 2020 and 2021 (the year following fall-applied treatments).

Trial Year	Treatment ^z^	Yield (t/ha)	At-harvest		
			Sugar (%)	RWS (kg/t) ^y^	RWSH (t/ha) ^x^
2019-20	Control	9.6	14.1	101.6	1.0
	Plow	9.7	14.9	109.1	1.1
	Heat (1.6 kmph)	7.7	14.3	103.9	0.8
	Desiccant	7.8	14.5	105.6	0.8
	*SE*	*0.7*	*0.2*	*1.9*	*0.1*
	*P-values*	*0.0908*	*0.1206*	*0.0797*	*0.0904*
	*LSD*	*-*	*-*	*-*	*-*
2020-21	Control	5.7	15.2	110.6	0.6
	Plow	5.7	14.8	107.3	0.6
	Heat (1.6 kmph)	7.2	14.9	108.1	0.8
	Desiccant	3.9	15.1	110.1	0.4
	Heat (4.8 kmph)	4.3	15.3	111.8	0.5
	*SE*	*1.0*	*0.2*	*1.7*	*0.1*
	*P-values*	*0.0783*	*0.3456*	*0.2598*	*0.1152*
	*LSD*	*-*	*-*	*-*	*-*
2021-22	Control	12.2	14.0	100.7	1.2
	Heat (4.8 kmph)	14.2	14.0	100.3	1.4
	*SE*	*4.5*	*0.2*	*1.8*	*0.5*
	*P-values*	*0.6358*	*0.9914*	*0.9154*	*0.6505*
	*LSD*	*-*	*-*	*-*	*-*

^z^ Non-treated control, plow with a 3-m tandem disc set to invert soil 15 cm. immediately post-harvest, heat treatment using a propane-fueled burner (Multi-Trail Enterprises LLC) calibrated to heat foliage to 649-871°C at 1.6 kmph and 4.8 kmph prior to defoliation, and a desiccant (Sharpen 0.07 L/ha, methylated seed oil 1% v/v, ammonium sulfate 2037 g/L) applied seven days pre-harvest.

^y^ Kilograms recoverable white sugar per metric ton of fresh beets (RWS).

^x^ Metric tons of recoverable white sugar per hectare (RWSH) calculated for each replicate using the following equation: RWSH (metric ton/hectare) = RWS (kg/metric ton) × Total Yield (metric ton/hectare) ÷ 1000; treatment means across four replicates are shown.

## 4 Discussion

Fall heat treatment of CLS-infested sugarbeet foliage (at temperatures 649-871°C) consistently reduced *C. beticola* sporulation *in planta* immediately following burner application. Reductions in isolations from leaves were not consistently observed, however, frequencies were low due to inhibition of *C. beticola* growth by competition of abundant soil microorganisms and potential antagonists. The sporulation results support previous reports that high temperatures were lethal to *C. beticola* (Pool and McKay, 1916). Following either fall- or spring-applied heat treatments, CLS levels in the subsequent sugarbeet crop were also reduced with consistent decreases in AUDPC. These observations indicate initial reductions in *C. beticola* viability and overwintering survival further impacted subsequent conidia production and in-field CLS severity. Burning for “thermosanitation” has been shown to reduce inoculum and incidence of plant pathogens in forestland, fruit crops, cotton, sugarcane, and grain crops (Hardison, 1976). In particular, burning stubble was shown to effectively control *Claviceps purpurea* (causal agent of ergot) and *Gloeotinia temulenta* (causal agent of blind seed disease) in grass seed crops (Hardison, 1980). It is important to note that the method tested in this study did not burn the leaves (**Supplementary Figure 1C**) or yield large amounts of smoke, which are problems with burning for disease management. Moreover, temperatures at or above 121°C were found to reduce *Rhizoctonia oryzae-sativae* sclerotia survival *in vitro* (Lanoiselet et al., 2005). Together, these studies demonstrated that high temperatures for brief periods of time can affect fungal survival, reproduction, and subsequent disease development, which is likely to be a factor for *C. beticola* as well, even in leaf tissue.

Beyond the overwintering impacts, the fall-applied heat treatment at 1.6 kmph consistently resulted in significant reductions in early-season spore levels. No notable reductions were achieved using the plow or desiccant treatments or using heat treatment at 4.8 kmph. The 4.8 kmph heat treatment did not significantly reduce early-season *C. beticola* levels, which indicates that a longer heat exposure, as would be provided by the 1.6 kmph treatment, may provide more effective and more consistent inoculum management. High temperatures over a certain period of time are typically needed to eliminate a particular pathogen, though this is temperature and pathogen dependent (Suárez-Estrella et al., 2003; Lanoiselet et al., 2005; Jung et al., 2009). Significant impacts on *C. beticola* were observed in the current study after an estimated less than 1 second exposure per plant (approx. 5-second exposure per meter) to temperatures of 649-871°C.

The results of this study also demonstrated the potential for both heat treatments, along with the already well-established plow treatments, to increase leaf degradation over the winter, which may also contribute to reductions in subsequent inoculum levels. Plowing has long been known to reduce CLS survival (Townsend, 1914). Buried residue has been shown to decompose faster than residue left on the soil surface (Nagel, 1938; Solel, 1970; Ruppel, 1986; Pereyra et al., 2004; Khan et al., 2008) with potential to reduce pathogen survival. Similarly, buried wheat residue decomposition to less than 2% after 24 months was associated with 50% reductions in colonization of *Fusarium graminearum* (syn. *Gibberella zeae*) inoculum, the disease-causing agent of Fusarium head blight (Pereyra et al., 2004). The current study demonstrated that the plow treatment (depth of 15 cm) increased sugarbeet leaf residue degradation numerically by 5 to 10 percentage points compared to the control at 90- and 135-DPH in 2020-21, with corresponding significant reductions in *C. beticola* sporulation observed up to 90-DPH. Notably, the fall heat treatment at 1.6 kmph resulted in comparable increases in residue degradation and reductions in *C. beticola* sporulation. Overall, significant impacts of tillage and heat treatment on sugarbeet leaf residue (and *C. beticola* viability) were achieved after only 3 to 4 months. These findings support the potential use of heat treatment as a viable alternative to plowing, which is more compatible with current recommendations for limited tillage (Hao et al., 2001; Tzilivakis et al., 2005).

While effective at managing crop residues, studies also have shown that soil-disturbing management strategies, such as plowing and tillage, can have undesirable impacts on soil structure, nutrient levels, and microbial populations (Hungria et al., 2009; Miura et al., 2015; Xue et al., 2018). Further tillage consequences can include reduced soil fertility, loss of soil structure and porosity, as well as reduced soil organic matter and beneficial soil organisms (Rieke et al., 2022). Conventional tillage also may increase soil erosion, pesticide and nutrient runoff, and emission of greenhouse gases (Withers and Lord, 2002; Cerdan et al., 2010; Smith et al., 2016). Minimum tillage or no-till has been a recommended practice globally, especially in areas with limited water (Hao et al., 2001; Tzilivakis et al., 2005). In sugarbeet, root and sugar yields were not significantly altered from conventional tillage to no-till management systems and an estimated $111/ha could be saved through decreases in fuel, labor, and total machine costs under a no-till system (Afshar et al., 2019). While plow and tillage treatments have been shown to increase leaf degradation and reduce CLS pressure in numerous experiments (see above) including the current study, similarly effective alternative practices for beet residue management, such as heat treatment, would be valuable for the industry. The current study supports the need for additional management tools for conventional and organic systems with the fall- and spring-applied heat treatments showing greater promise as an integrated pest management strategy compared to tillage.

Despite consistent reductions in *C. beticola* sporulation over the winter following plow treatments, there were no observable differences in early-season *C. beticola* conidia levels detected by sentinel beets and inconsistent impacts on AUDPC between trial years and locations. In Michigan, variability in efficacy of the plow treatment could be attributable to differences in winter conditions. Total precipitation from September to April was 190 mm more in 2019-20 (**Supplementary Figure 3A**) than 2020-21 (**Supplementary Figure 3B**); winter soil temperature during the same interval was slightly lower in 2020-21 compared to 2019-20 (Enviroweather, n.d). Differences in soils and environments are known to impact *C. beticola* survival. Changes in soil moisture, temperature, and microbial communities could all affect residue decomposition (Homet et al., 2021). Due to the variability in plow and tillage impacts observed here, the heat treatment may offer targeted and more reliable effects on *C. beticola* and subsequent CLS management, but this will need further testing in varied environments.

Compared to tillage treatments, heat treatment was expected to minimize effects in the soil profile (Hardison, 1976; Rahkonen et al., 1999). For example, minimal soil heating occurs during open grass fires and there is very little impact on the soil surface by flame treatment (Hardison, 1976). Furthermore, propane heat treatment at 100 kg/ha had little effect on microbial biomass in the upper soil profile where temperature increases of 4.0°C and 1.2°C were observed at 5- and 10-mm depths, respectively (Rahkonen et al., 1999). Thus, heat treatments present minimal threat to the soil microbial communities. In the current study, both heat treatments were briefly applied, with the fall treatment applied above the soil at canopy level and considerable reductions in temperature at and below the soil surface, further limiting the temperature impact on the soil structure and microbial communities. No negative impacts of the heat treatment were observed on sugarbeet yield or quality, however, further studies of potential impacts on microbial communities would be necessary. Notably, the heat treatment did not negatively affect sugar levels. Yields across all trial years in the Michigan tests were limited and below industry standards of approximately 64 MT/ha (USDA-NASS (United States Department of Agriculture, National Agricultural Statistics Services), 2019), likely due to back-to-back planting of sugarbeets in two consecutive years (research tool only), minimal pre-season nutrient inputs, and no fungicide applications to manage diseases, including CLS, Rhizoctonia root and crown rot, and others present in the region. Current conclusions, therefore, focus on the potential of each practice to manage *C. beticola* survival in sugarbeet residues and the reductions in subsequent CLS pressure.

The cost of heat treatment is relatively inexpensive, compared to time and labor, for weed management (Crop watch, 2015; Flame Engineering Inc, n.d) or application costs for fungicide applications, as well as additional soil compaction if repeatedly entering the field for ground applications. At 1.6 kmph, the propane-fueled, tractor-mounted heat treatment used for the fall-applied field study used approximately $300/ha in propane (output of 465 L/ha), based on the ten-year average for U.S. residential propane prices of $0.63/L (ten-year minimum: $0.47; maximum: $0.97) (US DOE-EIA (United States Department of Energy - Energy Information Administration), 2022). In Michigan, sugarbeet growers can spend between $350-600/ha for one season of foliar disease management (7-8 fungicide applications each $50-75/ha), based on estimated prices of standard products and dependent on variety tolerance and chemical program used (*personal communication*, C. Guza, Michigan Sugar Company, 2022); similarly, in Minnesota, sugarbeet growers can spend $300-375/ha. The heat treatment alone was not sufficient for a commercially acceptable level of disease management in Michigan or Minnesota, but it could delay or reduce the number of fungicide sprays in the growing season. Observed reductions in CLS severity following spring and fall heat treatments were sustained over several weeks of the sugarbeet growing season (up to four to eight weeks after initial detection). These reductions suggest that integration of a heat treatment into an existing management program has potential to replace up to two to four early-season fungicide applications (e.g., up to $300/ha in an 8-application foliar program in Michigan). Localized research on how heat treatment would fit into a larger CLS management program is needed before such technology could be adopted by beet growers.

Previous studies have demonstrated that propane-fueled flame management can be a useful strategy for weed and disease control for both organic and conventional production systems (Hardison, 1976; Hardison, 1980; Lanoiselet et al., 2005). Furthermore, heat treatments offer a potential integrated option where there are documented fungicide resistance issues, as heat exposure is a completely different mode of disease management. Pesticide use can lead to resistance development in weed and pathogen pests (OEPP/EPPO, 1999; Leadbeater et al., 2019), and reduced sensitivity to multiple fungicide classes has been observed in widespread *C. beticola* populations. Considering the application and product costs associated with chemical control, heat treatments may be a beneficial tool to use in beet growing regions where CLS epidemics are frequent, severe, and where fungicide use or efficacy is limited due to resistance in *C. beticola* populations. Heat treatment could further be of interest where fungicide resistance and residue management are a concern for other *Cercospora* species, such as *C. zeae-maydis* (gray leaf spot of corn), *C. kikuchii* (leaf blight and purple seed stain of soybean), *C. sojina* (frogeye leaf spot of soybean), *C. arachidicola* (peanut), and *C. carotae* (leaf spot of carrot) (Farr et al., 1989).

Results from Michigan and Minnesota field trials (2019-22) consistently indicate the use of a foliar heat treatment at-harvest or pre-planting has the potential to significantly reduce inoculum and CLS levels for the following growing season. A heat treatment could be useful for regions annually impacted by CLS and might be used to reduce fungicide applications, as well as mitigate fungicide resistance development. The use of foliar heat treatment is a novel control strategy for managing CLS as an additional tool in an integrated pest management program.

## Data availability statement

The raw data supporting the conclusions of this article will be made available by the authors, without undue reservation.

## Author contributions

AH was the Ph.D. graduate student responsible for conducting and coordinating the Michigan field, laboratory, greenhouse research, conducted the data analysis, and was the lead author in the development of this article. DB and TW were responsible for operation and maintenance of the burner equipment in Michigan and DB developed the initial hypothesis for heat treatment applications. SR assisted with the Michigan field, laboratory, and greenhouse components of this work. CB supported this research in the maintenance and harvest of the Michigan trials as well as participating with yield and quality data collection. DM and MB were responsible for the Minnesota field research and data collection components of this work. LH advised and contributed to the conceptual design of the work. JW is the corresponding author for this work responsible for the conceptual design as well as revision and final approval of the article. All authors contributed to the critical revision of this article. All authors contributed to the article and approved the submitted version.
