# Supplementary material for: An in-field heat treatment to reduce Cercospora beticola survival in plant residue and improve Cercospora leaf spot management in sugarbeet
Source: Front Plant Sci. 2023 May 9;14:1100595. doi: 10.3389/fpls.2023.1100595 (PMC10204640; doi:10.3389/fpls.2023.1100595)
Supplement: Supplementary file 1 [file DataSheet_1.docx]

Supplementary Material

An in-field heat treatment to reduce *Cercospora beticola* survival in plant residue and improve Cercospora leaf spot management in sugarbeet

Alexandra P. Hernandez^1^, Daniel M. Bublitz^3^, Thomas J. Wenzel^3^, Sarah K. Ruth^1^, Chris Bloomingdale^1^, David C. Mettler^4^, Mark W. Bloomquist^4^, Linda E. Hanson^2^, and Jaime F. Willbur^1*^

^1^Potato and Sugarbeet Pathology, Michigan State University, Department of Plant, Soil and Microbial Sciences, East Lansing, Michigan, USA

^2^Sugarbeet and Bean Research Unit, United States Department of Agriculture – Agricultural Research Services, East Lansing, Michigan, USA

^3^Michigan State University Extension and Sugarbeet Advancement, Frankenmuth, Michigan, USA

^4^Southern Minnesota Beet Sugar Cooperative, Renville, Minnesota, USA

*** Correspondence:** willbur1@msu.edu

Supplementary Table 1. Early-season Cercospora leaf spot (CLS) lesion observations on live spore traps^z^ (sugarbeet plants) in Michigan field studies sampled in 2020, 2021, and 2022 (the year following fall-applied treatments).

| Year | Week | Placed in the field | Removed from the field | Sampling duration (days) | Days after planting |
| --- | --- | --- | --- | --- | --- |
| **2020 ^y^** | **1** | **26-May** | **2-Jun** | **7** | **39-46** |
| **2020** | **2** | **2-Jun** | **9-Jun** | **7** | **46-53** |
| 2020 | 3 | 16-Jun | 23-Jun | 7 | 60-67 |
| 2020 | 4 | 23-Jun | 30-Jun | 7 | 67-74 |
| 2020 | 5 | 30-Jun | 7-Jul | 7 | 74-81 |
| 2020 | 6 | 7-Jul | 14-Jul | 7 | 81-88 |
| **2020** | **7** | **14-Jul** | **21-Jul** | **7** | **88-95** |
| 2021 | 1 | 14-May | 21-May | 7 | 7-14 |
| **2021** | **2** | **1-Jun** | **8-Jun** | **7** | **25-32** |
| **2021** | **3** | **15-Jun** | **22-Jun** | **7** | **39-46** |
| **2021** | **4** | **29-Jun** | **6-Jul** | **7** | **53-60** |
| **2022** | **1** | **17-May** | **24-May** | **7** | **18-25** |
| **2022** | **2** | **24-May** | **31-May** | **7** | **25-32** |
| 2022 | 3 | 1-Jun | 8-Jun | 7 | 33-40 |
| **2022** | **4** | **15-Jun** | **22-Jun** | **7** | **47-54** |

^z^ Live spore traps were placed in the center of each plot and consisted of four sentinel beets of USDA germplasm F1042 (USDA Agricultural Research Service, 2017) to detect viable *Cercospora beticola* airborne spores (Bublitz et al., 2021).

^y^ Bolded weeks were included in the results and analyses. No or low detections in weeks not shown due to low spore concentrations or other external insect or drought stress factors.


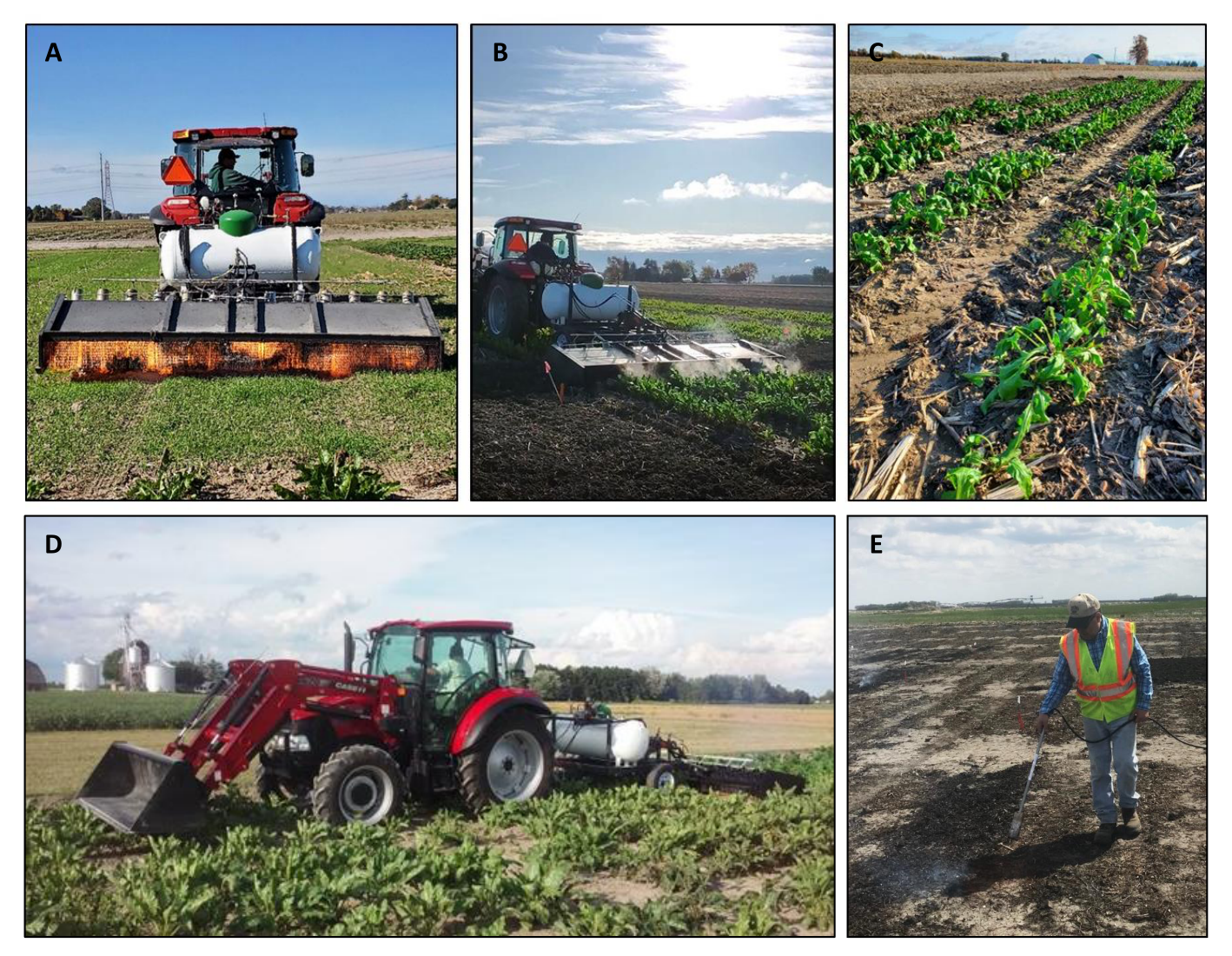


Supplementary Figure 1. **(A-B, D)** Custom designed 3.25-m wide propane-fueled, tractor-mounted shield burner (Multi-Trail Enterprises LLC) used for application of the fall heat treatments calibrated to heat foliage to 649-871°C. **(C)** Sugarbeet foliage immediately following 1.6 kmph heat treatment. **(E)** Handheld Flame King Heavy Duty Propane Torch Weed Burner (Pico Rivera CA 90660) used to apply spring heat treatments.


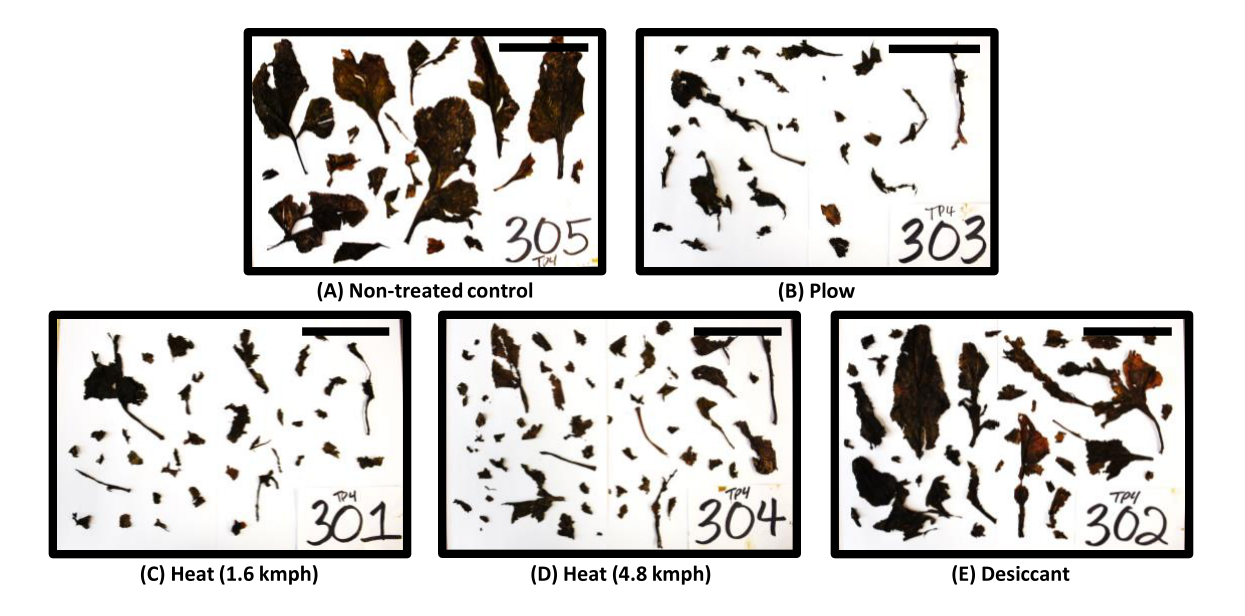


Supplementary Figure 2. Sugarbeet leaf degradation in overwintered samples collected 135-days post-harvest (DPH) from one representative replicate of field plots treated with the following: **(A)** non-treated control, **(B)** plow with a 3-m tandem disc set to invert soil 15 cm. immediately post-harvest, heat treatment using a propane-fueled burner (Multi-Trail Enterprises LLC) calibrated to heat foliage to 649-871°C at **(C)** 1.6 kmph and **(D)** 4.8 kmph prior to defoliation, and **(E)** a desiccant (Sharpen 0.07 L/ha, methylated seed oil 1% v/v, ammonium sulfate 2037 g/L) applied seven days pre-harvest (scale bar = 10 cm). Field plots were located at the Saginaw Valley Research and Extension Center in Frankenmuth, MI; all treatments were applied in the fall prior to or immediately following sugarbeet harvest in 2020 and leaf sampling continued through 135-DPH in 2021. Samples consisted of eight mid-canopy leaves which were collected at 0-DPH, weighed, and returned to the field in mesh bags to overwinter at representative soil depths for 45-, 90-, and 135-DPH.





Supplementary Figure 3. Daily maximum (solid line) and minimum (dotted line) soil temperatures at a depth of 5 cm and total precipitation (solid bars) measured from **(A)** September 1, 2019 to April 1, 2020 and **(B)** September 1, 2020 to April 1, 2021 at the Saginaw Valley Research and Extension Center in Frankenmuth, Michigan. Data were collected from the Richville/Frankenmuth weather station supported by Michigan State University and the Enviroweather project.
